# 3-D pathological analysis of varicose microvascular vessels in sessile serrated lesions seen on endoscopy

**DOI:** 10.1055/a-2886-4405

**Published:** 2026-06-16

**Authors:** Yosuke Iwasa, Fumikazu Koyama, Kohei Morita, Kosuke Fujimoto, Yuichi Teramura, Akihiko Yoshizawa, Masayuki Sho

**Affiliations:** 1 Surgery 12967 Nara Medical UniversityKashiharaNaraJapan; 2 Pathology 723194 Nara Medical University Department of Diagnostic PathologyKashiharaNaraJapan; 3 Clinical Research Endoscopy System Division and Medical System Business Division 34778 Fujifilm CorporationMinatoTokyoJapan

We have clarified the vascular structure of colorectal lesions by contrasting three-dimensional (3D) reconstructed images of pathological slides using SYNAPSE VINCENT with *in vivo* endoscopic images. 1​ We showed that the blood vessels visible in magnified blue light imaging (BLI) images are capillaries located within 80 μm of the surface. 2​

Sessile serrated lesions (SSLs) exhibit varying macroscopic and vascular morphologies, necessitating differentiation from hyperplastic polyps due to their malignant potential. 3​ Characteristic findings of SSLs include varicose microvascular vessels (VMVs), which are considered useful in differentiating them from hyperplastic polyps, but their histopathological basis are unclear. 4​ This study aims to clarify the anatomy of VMVs by comparing 3D reconstructed images of a part of SSLs containing VMVs with BLI.

**Fig. 1:**
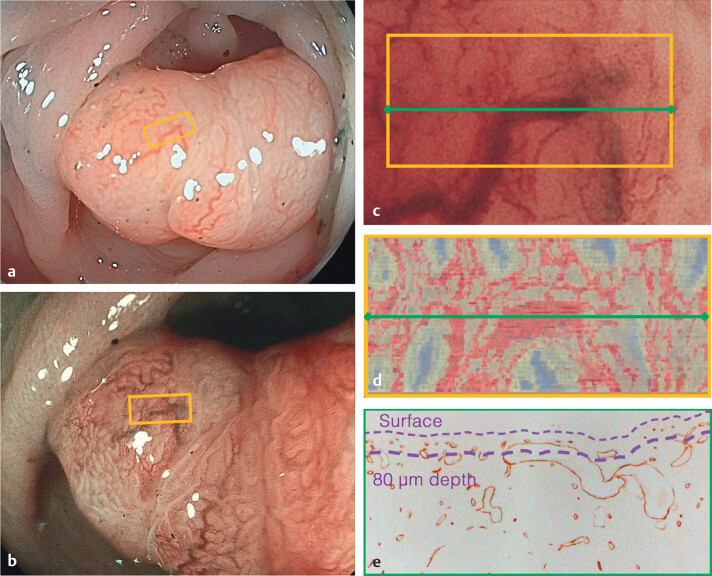
Comparison between the 3D reconstructed histological image with the corresponding 2D histological tissue digital microscopic image and the horizontal plane endoscopic images. The yellow squares show the same area, and the green arrows show identical capillaries. ( **a** ) Low magnification with a white light image. ( **b** ) Low magnification imaging with BLI. ( **c** ) The CD34-stained histopathological image. The green line where the varicose microvascular vessel is running horizontally. ( **d** ) The top view of the 80-μm slice 3D reconstructed image. ( **e** ) Histopathological slide with CD34 staining. The center of the histopathological image corresponding to the varicose microvascular vessel lying in the endoscopic image.

Endoscopic and pathological vascular images of two SSL patients are shown. An outline of this process is presented in Fig. 1 . The video shows the process of comparing 3D reconstruction of histopathology with actual BLI and analyzing the corresponding vascular depth from the mucosal surface ( Video 1 ). First, a 3D vascular model of the entire SSL was constructed. The vascular structure was maintained in the SSL, from the deep mucosa, running up and down along the sides of the glands, and forming capillary plexuses at the surface. One-to-one correspondence between the BLI and the vascular network of the 3D model revealed the anatomical location of the VMVs. All three VMVs in the two cases were dilated capillaries within the mucosa and were located within approximately 80 μm from the surface.

**Video 1** The identification of varicose microvascular vessels in sessile serrated lesions by comparing endoscopic images and 3D reconstructed pathological images using the Synapse VINCENT software program (Fujifilm Co., Tokyo, Japan).


It was confirmed that the capillary network and VMVs observed by magnified BLI is limited to within 80 μm from the surface. Comparing the endoscopic images of observed lesions with the pathological images of the corresponding areas improves the accuracy of endoscopic diagnosis.

Endoscopy_UCTN_Code_TTT_1AQ_2AB
